# Exploring the growing forest musk deer (*Moschus berezovskii*) dietary protein requirement based on gut microbiome

**DOI:** 10.3389/fmicb.2023.1124163

**Published:** 2023-03-09

**Authors:** Ruiguang Gong, Shengjie Song, Yaotian Ai, Shuhui Wang, Xianggui Dong, Zhanjun Ren, Hui Xie, Benmo Jiang, Lixia Zhao

**Affiliations:** ^1^College of Animal Science and Technology, Northwest A&F University, Yangling, Shaanxi, China; ^2^Qinba Ecological Protection Center of Chenggu County, Baoji, Shaanxi, China; ^3^Baoji Fengchun Forest Musk Breeding Base, Baoji, Shaanxi, China; ^4^Shaanxi Shenglinyuan Biotechnology Co., Ltd., Baoji, Shaanxi, China

**Keywords:** *Moschus berezovskii*, crude protein, 16S rRNA, gut microbiota, growth performance

## Abstract

It is necessary to assess the appropriate dietary protein level of the forest musk deer (FMD), as nutritional needs are unclear. The microbiome in gastrointestinal tracts plays an important role in regulating nutrient utilization, absorption and host growth or development. Thus, we aimed to evaluate growth performance, nutrient digestibility and fecal microbiome of growing FMD supplied with different protein levels of diets. Eighteen 6-month-old male FMD with an initial weight 5.0 ± 0.2 kg were used in a 62-day trial. The animals were randomly distributed to three groups, the dietary crude protein (CP) level was 11.51% (L), 13.37% (M), and 15.48% (H). The results showed that the CP digestibility decreased as dietary CP level increased (*p* < 0.01). Compared with group L and H, FMD in M group has higher average daily gain, feed efficiency and neutral detergent fiber digestibility. For the fecal bacterial community, the percentage of Firmicutes was increased, Bacteroidetes was decreased and the diversity of microbiota significantly reduced (*p* < 0.05) with the increasing of dietary protein. The proportion of *Ruminococcaceae_005*, *Ruminococcaceae_UCG-014* and *uncultured_bacterium_f_Lachnospiraceae* were significantly increased wtih rising CP, the proportions of *Bacteroides* and *Rikenellaceae_RC9_gut_grou*p were significantly decrease nevertheless at the genus level. The higher abundance of *f_Prevotellaceae* and *g_Prevotellaceae_UCG_004* were found at M group by LEfSe analysis. The relative abundance of *uncultured_bacterium_f_Ruminococcaceae* was positively correlated with the average daily gain and feed conversion ratio (*p* < 0.05), whereas *Family_XIII_AD3011_group* was negatively correlated with feed conversion ratio (*p* < 0.05). The UPGMA tree showed L and M groups were closer in clustering relationship, while H group was clustered separately into a branch, which indicated that the bacterial structure had changed greatly with protein level increased from 13.37 to 15.48%. Overall, our results indicated that the optimum dietary CP for the growing FMD was 13.37%.

## 1. Introduction

Forest musk deer (*Moschus berezovskii*), one of the small ruminants, belongs to a special economic animal in China. Musk, which secreted by male forest musk deer (FMD), is a valuable resource in the traditional Asian medicine and the international perfume industry (Wang et al., 2022). The number of wild FMDs was sharply declined as the overhunting for the musk. In 2008, wild FMD was listed as an endangered species by the International Union for Conservation of Nature (Wang and Harris, 2008). In China, captive breeding the FMD has become an important way for obtaining the musk (Meng et al., 2006). In this way, artificial breeding not only meets the demand of the musk for drug synthesis and perfume making, but also protects the population of wild FMD from the further decline. However, the captive FMDs are still fed in a relatively primitive way and the food items and composition are vary greatly from farms (Wang et al., 2015). So, limited feeding standard and incomplete nutritional requirements limit the growth performance and health status of the FMD. Better body condition of FMD during the growing phase plays an important role in a series of physiological processes, such as rut, breeding and musk secretion. In local feeding farm, besides leaves, the feedstuffs for FMD are simple mixture of the chopped pumpkin, carrot and concentrate, and the concentrate mainly consisted of corn meal, soybean meal and bran, with different proportions in different farms. But the moldy leaves or rotten vegetables constantly lead to the intestinal diseases (Wang et al., 2013), which further induced the defective of the FMD normal growth in breeding industry (Zhu et al., 2012; Li et al., 2017; Yang et al., 2021).

Dietary protein levels are essential factors that affect the growth, development and health conditions of animals (Schroeder et al., 2006). Therefore, exploring the dietary protein requirement of FMD is first. Chinese management policy for the FMD breeding industry is very strict, slaughter or injury the FMD are strictly forbidden, so obtaining the samples for dietary protein demand research is restricted, even for the rumen fermentation sampling. Considering the pivotal role of the intestinal microbiota in the growth and development of the host (Schwarzer et al., 2016), studying the changes of fecal flora structure is an important way to monitor the health status of the forest musk deer (Hu et al., 2018; Liu et al., 2019). Changes in diet can alter the fecal microbiome of FMD. As the diet of FMD changes from consuming milk to eating plants, the richness and diversity of intestinal microbiota of young FMD increased significantly (Li et al., 2021). What’s more, previous found that dietary protein levels not only affect the growth performance of ruminants, but also play an important role in regulating the microbial structure of the digestive tract (Liu et al., 2022; Zhang et al., 2022).

The present study was carried out to explore the effects of different crude protein (CP) levels of TMR pellet feed on the growth performance, nutrient digestibility and fecal microorganisms of FMD during the growing phase, and provide reference data for the standardization of the nutritional needs for the captive FMD.

## 2. Materials and methods

### 2.1. Animals and diets

Eighteen 6-month-old FMD with a similar initial weight (5.0 ± 0.2 kg) were randomly divided into 3 groups consisting of 3 replicates with 3 FMD per replicate. The FMDs were fed with low CP diet (CP 11.51%, L), middle CP diet (CP 13.37%, M) and high CP diet (CP 15.48%, H), respectively. The experiment last for 62 days and all FMDs were fed at 9: 00 a.m. and 16: 00 p.m. All animals were housed individually and provided feed and water *ad libitum*. The health condition of each animal was closely monitored. The composition of the diets is presented in Table 1. The air-dried feed materials were crushed, then the pellet with the length of 8.0 mm-15.0 mm was made through the ring die granulator (compression ratio 1: 8, aperture 4.0 mm).

**Table 1:** Composition of the diets (%, as-fed basis).

Item	L	M	H
Ingredients			
Corn	29.14	24.86	21.88
Soybean meal	1.33	4.93	10.91
Wheat bran	7.15	4.95	6.02
Alfalfa hay	38.23	41.51	46.10
Mulberry branch bark powder	7.02	6.41	4.88
*Broussonetia papyrifera* silage	7.31	6.82	4.71
Beet pulp	3.82	5.02	0.00
Soybean oil	1.00	0.50	0.50
Permix^1^	5.00	5.00	5.00
Dry matter			
Digestible energy, MJ/kg	10.65	10.68	10.63
Crude protein	11.51	13.37	15.48
Crude fiber	12.74	12.39	11.99
Neutral detergent fiber	25.79	25.75	24.75
Lysine	1.70	1.70	1.70
Methionine + Cystine	0.60	0.60	0.60
Calcium	0.90	0.91	0.90
Available phosphorus	0.24	0.24	0.24

^1^Provided per kilogram diet: 8,000 IU of vitamin A; 500 IU of vitamin D_3_; 10 IU of vitamin E; 2 mg of vitamin K_3_; 50 mg of Fe (as ferrous sulfate); 4 mg of Cu (as copper sulfate); 2 mg of Mn (as manganese sulfate); 50 mg of Zn (as zinc sulfate); 0.2 mg of I (as KI); 0.2 mg of Se (as Na_2_SeO_3_).

### 2.2. Sampling and measurement

At 1 and 62 d of the trial period, body length gain (BLG), chest girth gain (CGG), body oblique length gain (BOLG) and weight gain were measured individually after overnight fasting (12 h), meanwhile, average daily feed intake (ADFI), average daily gain (ADG) and feed to gain (F: G) were calculated. Fresh fecal samples were collected on days 56–62 of the trial. Feces for apparent nutrient digestibility analysis were collected into valve bags, and 10 ml of 10% H_2_SO_4_ solution was evenly added to 0.1 kg of feces to fix fecal nitrogen. Other samples were snap-frozen in liquid nitrogen, and then stored at −80°C for further analysis.


BLG=Final body length−Initial body length

CGG=Final chest girth−Initial chest girth

BOLG=Final body oblique length−Initial body oblique length

ADFI=Total food intake during the test/Days of test

ADG=(final body weight−initial body weight)/Days of test

F:G=ADFI/ADG

### 2.3. Chemical analysis

All feed and fecal samples were assessed in triplicate with a 1-mm screen after finely ground. Samples were analyzed for dry matter (DM), CP, neutral detergent fiber (NDF) and acid detergent fiber (ADF), and apparent total tract digestibility of DM, CP, NDF and ADF were accumulated as previously described by Williams et al. (1962).

### 2.4. 16S rRNA gene profiling

The total genomic DNA of fecal bacteria was extracted using TIANGEN DNA Kit (TIANGEN, Beijing, China) according to the manufacturer’s instructions. The genomic DNA was used as a template for PCR amplification. Full-length (V1-V9) 16S rRNA was amplified by using the 27F (5′-AGR GTT YGA TYM TGG CTC AG-3′) and 1492R (5′-RGY TAC CTT GTT ACG ACT T-3′) primers and sample-specific barcodes. 10 μl mixture containing 40 ng of template DNA, primers, buffer, Taq polymerase and ddH_2_O. The following thermal cycling conditions was used: initial denaturation of 5 min at 95°C, 25 cycles of denaturation at 95°C for 30 s, annealing at 50°C for 30 s, extension at 72°C for 40 s, and a final extension at 72°C for 7 min. Fresh PCR products of almost-full-length 16S rRNA gene were purified by agarose gel and ligated into TA cloning kit of TOP 10 (Invitrogen, United States). Library construction was undertaken, then transformants were picked up randomly and the 16S rRNA gene was sequenced on the PacBio Sequel sequencing platform.

Circular consensus sequences (CCSs) were obtained by correcting the original subreads (SMRT link, version 8.0) according to minPasses of ≥5 and minPredictedAccuracy of ≥0.9. Lima v1.7.0 software was used to identify different samples according to the barcode. Cutadapt V2.7 (error rate 0.2) was used to identify the forward and reverse primers, and CCSs without a primer were discarded. Finally, CCS length was filtered, and sequences that did not meet the length threshold (16S: 1,200 bp to 1,650 bp; ITS: 300 bp to 1,000 bp) were discarded. Usearch v 10.0.240 (Edgar, 2013) was used to cluster CCSs at 97% similarity level, obtain operational taxonomic units (OTUs).

Venn diagram was drawn with VennDiagram version 1.6.20 (Chen and Boutros, 2011). Conduct taxonomic annotation of the OTUs based on Silva 132 (bacteria) (Quast et al., 2013) taxonomy databases. Query sequences were blasted against the reference database using the classify-consensus-blast methods, and the nonmatched sequences were further classified by classify-sklearn. Data on the community composition of each sample was obtained at various classification levels (phylum, class, order, family, genus, and species). The alpha diversity index of each sample was evaluated using QIIME2 software (Bolyen et al., 2019). Analysis of similarities (ANOSIM) was performed on unweighted UniFrac distance matrices for fecal communities and on Bray-Curtis similarities using Primer 6 software and the R package vegan. Principal coordinate analysis (PCoA) was performed using the mixOmics package in R (v3.2.1) based on the Bray-Curtis distances; non-metric multidimensional scaling was performed using the R package “Vegan.” Phylogenetic trees were constructed by unweighted pair-group method with arithmetic means (UPGMA). Spearman analysis was used to calculate the correlation coefficient between the different fecal microbiota and the growth performance (Wu et al., 2021). Microbiota-based biomarker discoveries were done with LEfSe using the online server^1^, and the LDA scores derived from LEfSe analysis (Segata et al., 2011) were used to show the relationship between taxon using a cladogram (circular hierarchical tree) of significantly increased or decreased bacterial taxa in the gut microbiota between groups.

### 2.5. Statistical analysis

An individual FMD was considered as an experimental unit in all statistical analysis. All data were analyzed using SPSS statistical package (IBM SPSS, Chicago, IL; 26.0 software). After checking the normality of the data distribution, the growth performance data were analyzed by one-way analysis of variance (ANOVA). All data were expressed as the mean with standard error (SEM), differences were considered as statistically significant at *p* < 0.05, and greatly significantly different at *p* < 0.01.

## 3. Results

### 3.1. Growth performance and nutrient digestibility

The effects of different dietary protein level on the growth performance and morphometric traits are shown in Table 2. The FMD receiving 13.37% CP diet had a higher ADG (*p* < 0.05) and lower F:G (*p* < 0.05) than that receiving dietary CP at 11.51%. Meanwhile, BLG significantly increased (*p* < 0.05) in M group compared with the L group.

**Table 2:** Effects of different dietary crude protein levels on growth performance and morphometric traits in growing forest musk deer.

Items	*L*	*M*	*H*	SEM	*p*-value
ADFI, g/d	186.47	182.45	183.82	1.350	0.494
ADG, g/d	8.20^B^	10.17^A^	9.44^A^	0.227	<0.001
F:G	22.79^A^	18.03^B^	19.51^B^	0.571	<0.001
BLG, cm	1.85^b^	2.23^a^	2.11^ab^	0.072	0.045
BOLG, cm	2.03	2.32	2.21	0.058	0.111
CGG, cm	1.13	1.18	1.18	0.027	0.701

A row with different lowercase letter superscripts were significantly different (*p* < 0.05); a row with different capital letter were highly significant different (*p* < 0.01). The same as below.

As presented in Table 3, the digestibility of DM in H group was higher (*p* < 0.05) than other groups. With the increase of CP in diet of FMD, the digestibility of CP significantly decreased (*p* < 0.01). FMD received 13.37% CP diet have the highest NDF digestibility (*p* < 0.01), but no difference was observed among groups on ADF digestibility (*p* > 0.05).

**Table 3:** Effects of different dietary crude protein levels on apparent nutrient digestibility in growing forest musk deer.

Items	*L*	*M*	*H*	SEM	*p*-value
DM (%)	59.91^b^	59.78^b^	60.74^a^	0.37	0.040
CP (%)	70.01^A^	67.43^B^	62.31^C^	0.86	<0.001
NDF (%)	57.45^B^	61.40^A^	60.84^A^	1.13	0.003
ADF (%)	31.05	32.12	32.63	0.34	0.154

A row with different lowercase letter superscripts were significantly different (*p* < 0.05); a row with different capital letter were highly significant different (*p* < 0.01).

### 3.2. Diversity of bacteria

**Figure 1:**
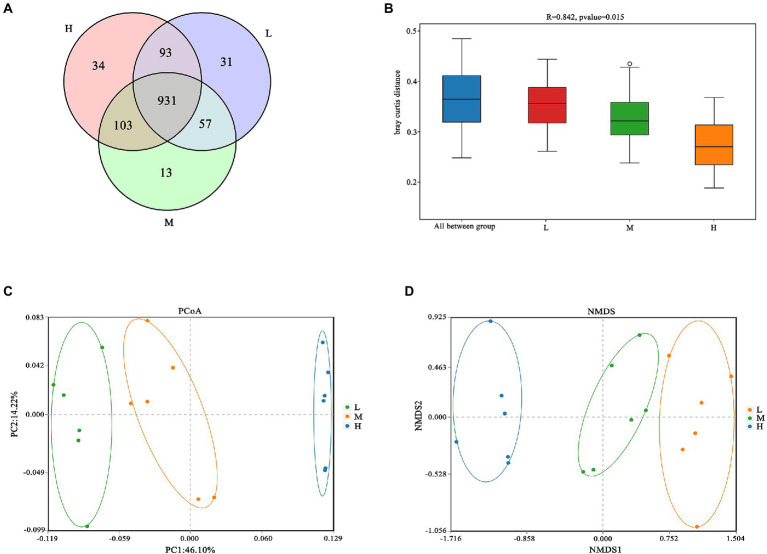
**(A)** Venn diagram; **(B)** Analysis of similarities (ANOSIM); **(C)** Principal coordinate analysis (PCoA); **(D)** Nonmetric multidimensional scaling (NMDS).

**Figure 2:**
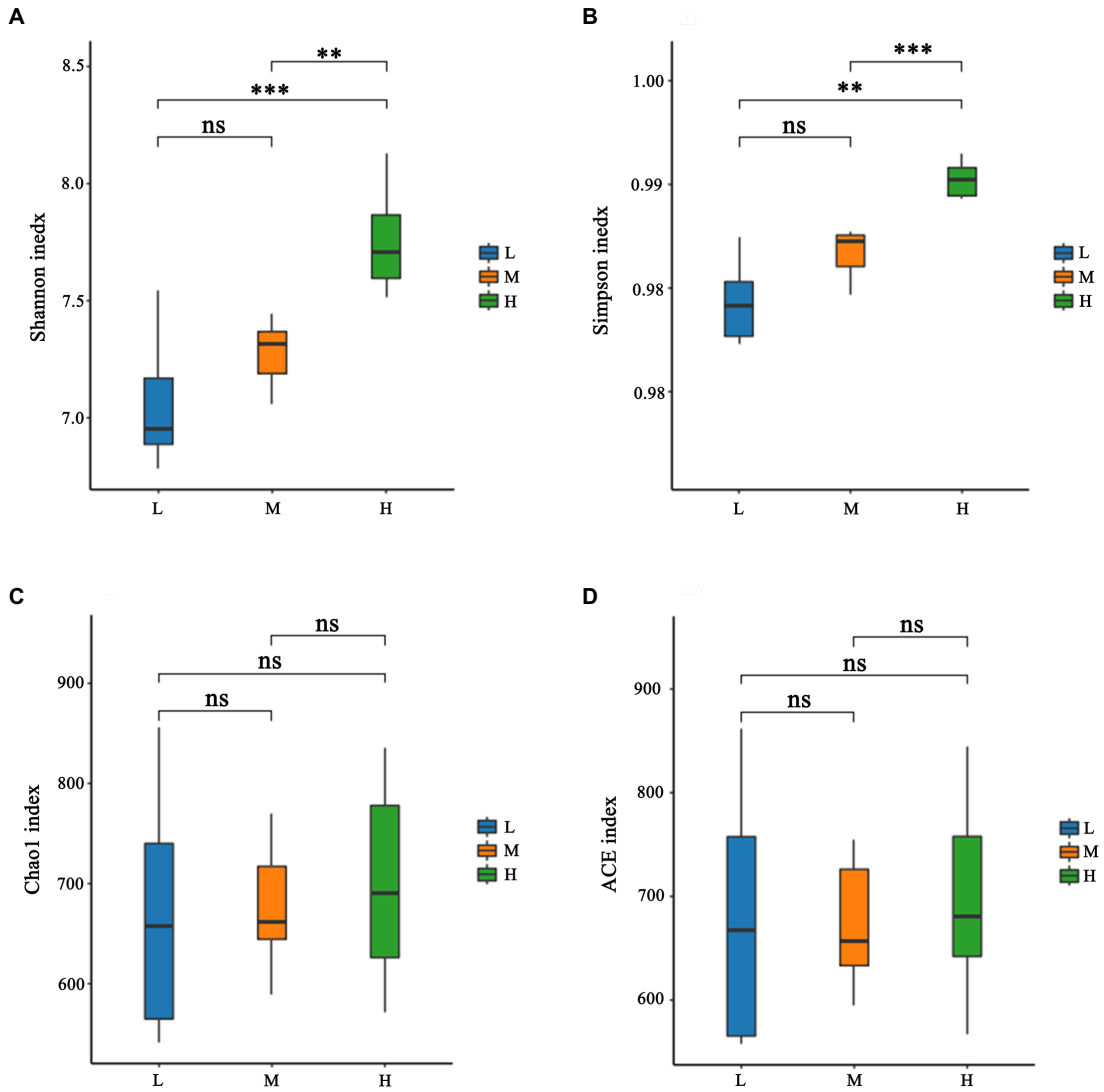
Alpha-diversity of fecal microbiota of forest musk deer fed diets with different levels of crude protein. **(A)** Shannon index; **(B)** Simpson index; **(C)** Chao1 index; **(D)** ACE index. ***, **, *, and ns were demonstrated *p* < 0.001, *p* < 0.01, *p* < 0.05, and not significant, respectively.

As shown in Figure 1A, a total of 931 OTU were shared in three groups; 34, 13 and 31 unique OTU were detected in group H, M and L, respectively. Further, the beta diversity on ANOSIM revealed that the three group’ fecal microbial communities were significantly different (*p* < 0.05, Figure 1B). There were significant differences of microbiota among three groups as shown in PCoA and NMDS (Figures 1C,D). Alpha-diversity indexes (Shannon, Simpson, Chao1 and ACE) were investigated and shown in Figure 2. Highest bacterial diversity (Shannon and Simpson indexes) were observed in FMD that fed with high CP dietary (*p* < 0.01), but the species richness values (Chao1 and ACE indexes) exhibited a similar trend.

### 3.3. Microbiota composition

**Figure 3:**
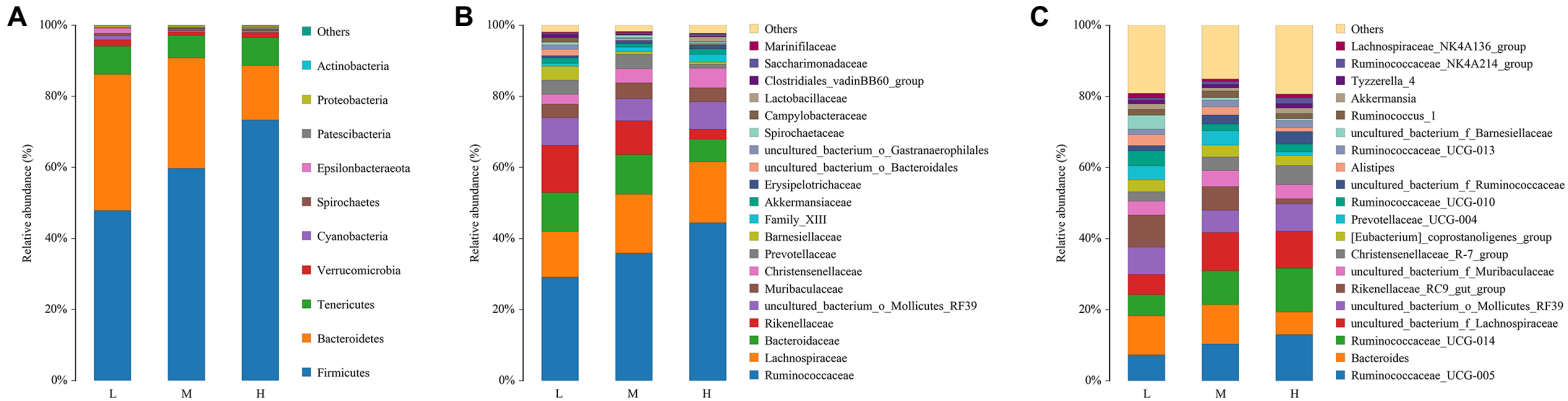
Relative abundance of bacterial composition in fecal contents at phyla **(A)**, family **(B)** and genus **(C)** levels among different dietary crude protein of growing forest musk deer. Each color represents one bacterium. The X-axis shows the different dietary treatments and the Y-axis shows the percentage of the bacteria.

The composition and abundance of fecal microbiota for FMD fed with different CP diets were analyzed. As shown in Figure 3A, the percentage of Firmicutes and Bacteroides were the highest at the phyla level. And the relative abundance of Firmicutes increased, Bacteroidetes decreased with the increase of dietary protein.

With the increase of crude protein level in dietary, Ruminococcaceae and Christensenellaceae increased, but Rikenellaceae reduced at the family level (Figure 3B).

As presented in Figure 3C, at the genus level, *Ruminococcaceae_005*, *Bacteroides, Ruminococcaceae_014*, *Lachnospiraceae*, *Mollicutes_RF39 and Rikenellaceae_RC9* were the major bacterial genera. Among these, the proportion of *Ruminococcaceae_005*, *Ruminococcaceae_014* and *Lachnospiraceae* increased with the increase of dietary protein, the proportions of *Bacteroides* and *Rikenellaceae_RC9* were decreased.

**Figure 4:**
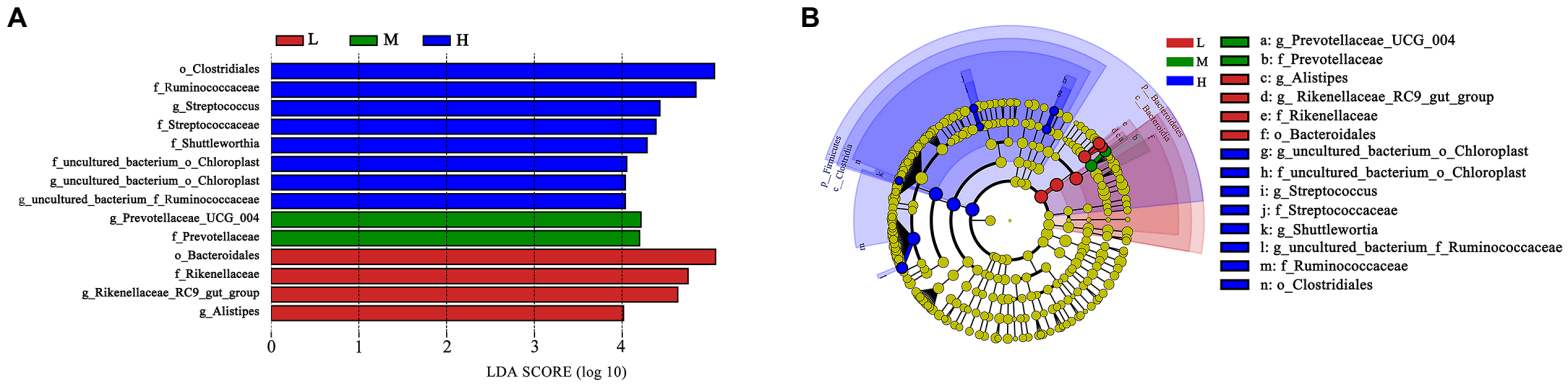
LEfSe analysis. **(A)** Different taxa microbes analysis in feces based on LEfSe method at 11.51% (L), 13.37% (M), and 15.48% (H) CP of FMD. The default parameters were LDA score > 2 and *p* < 0.05. Bacterium with red, green or blue colors mean that they are higher at L, M and H, respectively. **(B)** Taxonomic cladogram of forest musk deer fecal microbes at 11.51% (L), 13.37% (M), and 15.48% (H) CP levels. From inside to outside, different color circle represent different classification level (phylum, class, order, family and genus). The color of circles with letters mean that the bacterium was higher at specific crude protein levels of L, M or H groups are colored by red, green or blue, respectively.

Based on LEfSe analysis in Figure 4, the higher abundance of *g_uncultured_bacterium_o_Choroplast*, *f_uncultured_bacterium_o_Choroplast*, *g_Streptococcus*, *f_Streptococcaceae*, *g_Shuttleworthia*, *g_uncultured_bacterium_f_Ruminococcaceae*, *f_Ruminococcaceae* and *o_Clostridiales* were found in H group; the higher abundance of *g_Prevotellaceae_UCG_004* and *f_Prevotellaceae* were determinde in M group; and the higher abundance of *g_Alistipes*, *g_Rikenellaceae_RC9_gut_group*, *f_Rikenellaceae* and *o_Bacteroidale*s were determined in group L.

**Figure 5:**
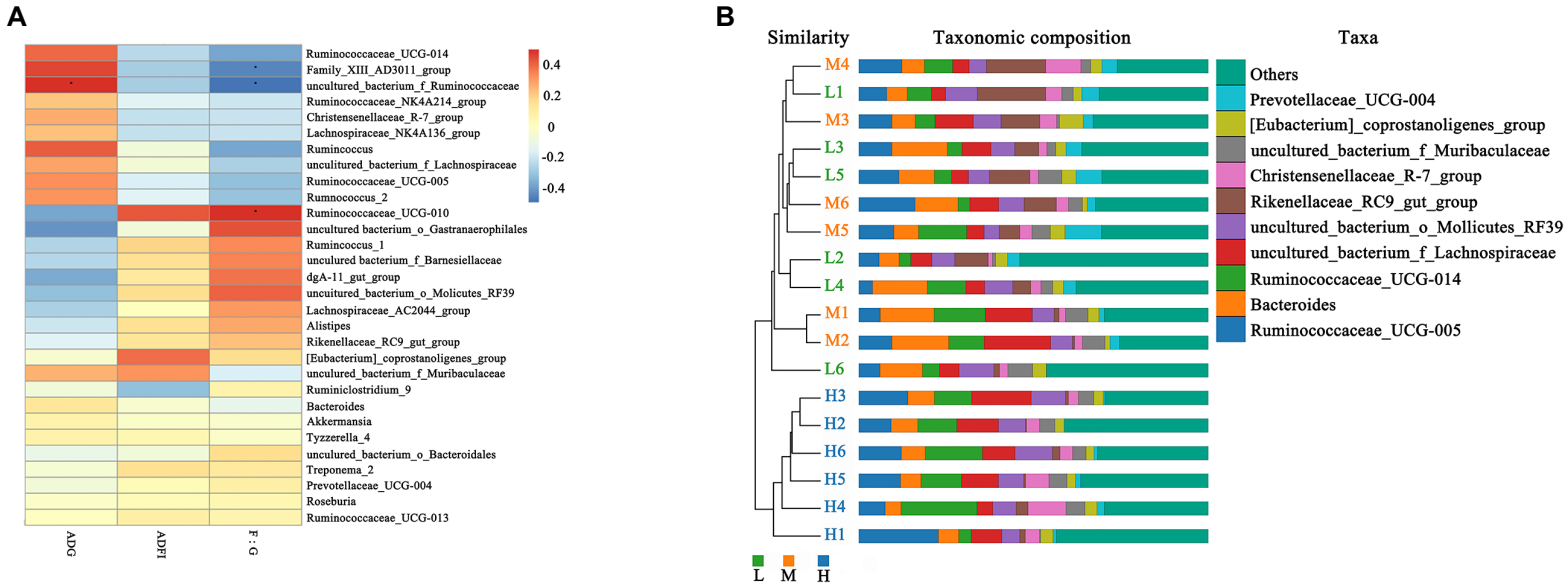
Correlation analysis and Unweighted Pair-group Method with Arithmetic Mean (UPGMA) analysis. **(A)** Spearman correlation analysis between differential genera and performance of growing forest musk deer. Significant correlations are noted by: ∗ 0.01 < *p* ≤ 0.05. **(B)** UPGMA based on OTU abundance at genus level for forest musk deer fed diets with different crude protein levels.

As shown in Figure 5A, the Spearman correlation matrix illustrated that the relative abundance of *uncultured_bacterium_f_Ruminococcacea*e was positively correlated with the ADG and feed conversion ratio of FMD (*p* < 0.05), only *Family_XIII_AD3011_group* was positively correlated with feed conversion ratio (*p* < 0.05). In contrast, *Ruminococcaceae_UCG-010* was negatively correlated with feed conversion ratio (*p* < 0.05). The UPGMA tree showed significant differences in the structure of fecal microbiota among three dietary treatments, L and M groups were closer in clustering relationship. This indicated that the high protein played an important role in the change of fecal microbial communities (Figure 5B).

## 4. Discussion

### 4.1. Changes in growth performance and nutrient digestibility of FMD Fed diets with different CP levels

Daily intake of food is required for metabolism in animals, and the feed intake is based on energy requirement. Lu and Potchoiba (1990) observed that dry matter intake decreased curvilinearly when dietary ME density increased in growing goat. In current study, although the 3 dietary protein levels were different, the energy values are similar, which is the reason why ADFI was not significantly different among the 3 diets despite the *ad libitum* feeding mode. Whereas in the growing phase of all animals, protein was required to make up the body. From the results of ADG, F:G and BLG data in this experiment, it can be concluded that compared with the 13.37 and 15.48% groups, the 11.51% protein level did not meet the nutritional needs of the growing FMD. During growing period of animals, bones are developed first, followed by muscles, and finally fat, namely the chest girth is lagging behind (Zhang, 2018). Therefore, compared with BLG and ADG, CGG is not significantly different in the 3 treatments. Dietary nutrient balance is vital to improve the feed conversion rate. Although the difference of F:G between M and H were not significant (*p* > 0.05), there was a trend of increase in the feed conversion ratio compared with H, which indicated that 15.48% CP level may be nutritional imbalance. Excessive dietary protein would reduce the growth performance and feed conversion rate. Jennings et al. (2018) noted that in the body, excess nitrogen is converted to urea in the liver and then excreted in the urine. Huntington and Archibeque (2000) calculated that 2.5 to 5% of whole body oxygen consumption was attributable to ureagenesis in the liver. Hales et al. (2013) reported that fed steers diet containing excess CP, whereas CP levels did not significantly affect N retention. Martin and Blaxter (1965) estimated the energy cost of synthesis of urea from ammonia to be 3.80 ± 0.57 Kcal/g of N in sheep. They noted that heat production, as a percent of metabolizable energy intake (MEI), increased and energy retention as a percent of MEI decreased as dietary CP concentration increased, suggesting that overall efficiency of energy utilization decreased with increasing CP content. These losses in energetic efficiency may potentially have a negative impact on animal growth and production efficiency. In addition, there is accumulation of excess N in the ruminal environment as ammonia which is no longer directed to the growth of microorganisms and therefore affects microbial efficiency as well as the optimization of degradation of available fodder (Pinheiro et al., 2020). This may explained the higher dietary protein level, the lower digestibility in the results.

Rumen microbes play an important role in uptake and digestion of the feed energy and nutrients, which help to convert the food into more valuable metabolites for the host animal. The growth and reproduction of microorganisms not only need to provide sufficient nitrogen, but also a sufficient carbon, carbon mainly comes from the decomposition of glucose, starch and cellulose. NDF is one of the main sources of carbon for rumen microorganisms (Xue et al., 2022). In this study, the digestibility of NDF in group L was significantly lower than that in M and H groups (*p* < 0.01). Cappellozza et al. (2021) also observed that protein supplementation to ruminants increased NDF digestibility. This may be due to the relative shortage of N supply in group L, which makes the microbial activity was weakened in group L, and finally leads to the reduction of the digestibility of NDF (Matthews et al., 2019). NDF is mainly composed of cellulose, hemicellulose, lignin and silicate, of which cellulose and hemicellulose can be decomposed by rumen microorganisms to produce volatile fatty acids (Miller et al., 2021), while ADF is mainly composed of lignin, in addition to less cellulose and ashes (Miranda et al., 2020). Compared with cellulose and hemicellulose, lignin is difficult to be decomposed and utilized by rumen microorganisms (Khonkhaeng and Cherdthong, 2019), therefore the digestibility of the 3 groups of ADF is not significantly different (*p* > 0.05).

The rumen and its microbiota play a particularly important role in the degradation of feedstuffs for FMD. A nutritionally balanced diet is important as it provides an environment that maximizes the growth and activity of these microbes. Rumen microorganism produces end products that are either utilized directly by the host or by other microorganisms as energy (Matthews et al., 2019). Further buffering is provided by ammonia produced during fermentation, which can then be used for microbial growth in the rumen. If the degradable protein in the diet is insufficient, it would cause a deficiency of ammonia nitrogen in the rumen, which further inhibits microbial growth and fiber digestion (Allen, 2000). In this experiment, the NDF levels of the 3 diets were similar, and with the increase of CP, the Shannon index and Simpson index increased, indicating that the rumen microbial diversity increased, which further suggested that the L group had insufficient N.

### 4.2. Changes in fecal microbiome of FMD Fed diets with different CP levels

The Venn analysis showed that the L, M and H groups had 31, 13 and 34 unique OTU, respectively, and shared 931 OTU. PCoA and NMDS showed that the structure of microflora changed regularly with the improvement of CP levels. This indicated that the microflora structure of M group is between L and H. Jiang et al. (2022) pointed out that higher α-diversity of gut microbiota probably benefits FMD adaption to different habitats. In the present research, group H has the highest α-diversity of fecal microbiota, this might indicate that the high protein of Group H has caused certain stress to the FMD, so that the intestinal bacteria have to make changes to cope with this stress. Firmicutes and Bacteroidetes had the hightest relative abundance in this study, which was consistent with the previous studies (Hu et al., 2017; Sun et al., 2020; Li et al., 2021; Su et al., 2022). Whereas the abundance of Firmicutes increased, and Bacteroidetes decreased with the increasing of dietary CP.

At the family level, the relative abundances of Ruminococcaceae and Christensenellaceae increased, while the relative abundances of Rikenellaceae and Barnesiellaceae decreased. In the current study, with the increase of CP levels, the relative abundance of fecal *Ruminococcaceae_UCG-005*, *Ruminococcaceae_ucg-014*, *uncultured_bacterium_f_Ruminococcaceae*, *Ruminococcaceae_NK4A214_group* and *Ruminococcaceae_UCG-013* increased at the genus level. Combined with ADFI, ADG, F:R and morphometric traits of growth FMD, it can be concluded that except for *Ruminococcaceae_UCG-013*, most genera in Ruminococcaceae were positively correlated with growth performance, and *uncultured_bacterium_f_Ruminococcaceae* being the most representative. In addition, *Family_XIII_AD3011_group* was also positively correlated with the growth performance of FMD during the growing phase (*p* < 0.05). Xie et al. (2022) noted that Prevotellaceae and Ruminococcaceae some members of these families can produce propanoic acid and butanoic acid, which have the effects of regulating the proliferation and differentiation of the intestinal stem cells. Ruminococcaceae plays critical roles in degrading a variety of polysaccharides and fibers, are generally the producers of short-chain fatty acids (Zhu et al., 2017).

As the main precursor for glucose synthesis, higher propionate levels are generally play beneficial effect on ruminant production (Bergman, 1990). Poudel et al. (2019) found Prevotellaceae possess the ability to increase ruminal propionate concentrations. The higher abundance of *f_Prevotellaceae* and *g_prevotellaceae_UCG_004* were found at M group as shown in taxonomic cladogram. It indicated that Prevotellaceae might plays an important role in promoting growth performance in FMD. According to the results of this experiment, it is speculated that the bacterium needs a suitable C:N ratio.

In present experiment, the growth performance of FMD in medium protein group is better than that H and L group. The feed conversion ratio and growth performance were improved linearly with an increased level of dietary CP (Prusty et al., 2016), but the growth performance would decreases if the protein exceeds the critical value. Excessive protein reduced protein utilization, and changed the structure of intestinal flora, promoted the proliferation of harmful intestinal bacteria (Chen, 2020). According to the fecal microbial community clustering results, at genus level, the H group was clustered separately into a branch, the clustering relationship between L and M groups is closer, indicating that the bacterial structure had changed greatly with protein level increased from 13.37% (M) to 15.48% (H), i.e., 15.48% dietary CP is excessive. Therefore, combined with growth performance and microbiome, the ideal dietary CP level was 13.37% for the growing FMD.

## 5. Conclusion

In conclusion, based on growth performance and microbiome, the relative abundance of *uncultured_bacterium_f_Ruminococcaceae*, *Family_XIII_AD3011_group*, *f_Prevotellaceae* and *g_prevotellaceae_UCG_004* could be used as a positive correlation indicators with growth performance, and *Ruminococcaceae_UCG-013*, might be a negative correlation discriminant index. Collectively, these findings also suggest optimal dietary CP levels of 13.37% for growing forest musk deer.

## Data availability statement

The datasets presented in this study can be found in online repositories. The names of the repository/repositories and accession number(s) can be found in the article/supplementary material.

## Ethics statement

The animal study was reviewed and approved by Northwest Agriculture & Forest University Animal Care and Use Committee.

## Author contributions

RG: conceptualization, methodology, data curation, and writing—original draft preparation. SS: investigation and data curation. YA: data curation. SW: sample collection and software. XD: visualization, investigation, and writing—review and editing. ZR: supervision and writing—reviewing, editing, project administration, and funding acquisition. HX, BJ, and LZ: resources. All authors contributed to the article and approved the submitted version.

## Funding

This work was supported by Key Research and Development Program of Shaanxi Province (K3180220029), and the Shaanxi Science and Technology Promotion Project (K3330218004 and K3130220004).

## Conflict of interest

LZ is employed by Shaanxi Shenglinyuan Biotechnology Co., Ltd.

The remaining authors declare that the research was conducted in the absence of any commercial or financial relationships that could be construed as a potential conflict of interest.

## Publisher’s note

All claims expressed in this article are solely those of the authors and do not necessarily represent those of their affiliated organizations, or those of the publisher, the editors and the reviewers. Any product that may be evaluated in this article, or claim that may be made by its manufacturer, is not guaranteed or endorsed by the publisher.

## Footnotes

^1^ https://huttenhower.sph.harvard.edu/galaxy/
